# Asynchronous optical coherence elastography and directional phase gradient analysis

**DOI:** 10.1117/1.JBO.30.12.124506

**Published:** 2025-09-19

**Authors:** Ginger Schmidt, Ryan McAuley, Brett E. Bouma, Néstor Uribe-Patarroyo

**Affiliations:** a Massachusetts General Hospital, Harvard Medical School, Wellman Center for Photomedicine, Somerville, Massachusetts, United States; b Institute for Medical Engineering and Science, Massachusetts Institute of Technology, Cambridge, Massachusetts, United States

**Keywords:** optical coherence elastography, optical coherence tomography, signal processing

## Abstract

**Significance:**

The stiffness and compliance of biological tissues are key properties that often change in the presence of pathology, yet current shear wave elastography approaches using optical coherence tomography (OCT) face limitations due to slow image acquisition, sensitivity to motion artifacts, and reliance on advanced hardware, hindering clinical translation.

**Aim:**

The aim is to develop and validate a practical, high-speed method for three-dimensional shear wave imaging compatible with standard OCT systems and wave propagation variability.

**Approach:**

We introduce a technique for the rapid, asynchronous acquisition of three-dimensional shear wave fields. Our technique operates at conventional acquisition rates and utilizes pairs of B-scans, similar to angiography scanning protocols. This approach significantly reduces motion sensitivity and enhances acquisition speed, even with much denser lateral sampling. In addition, we present a technique for estimating the shear wave number, termed directional phase gradient analysis. This method computes the phase gradient of the autocorrelation of the directionally-filtered, complex-valued shear wave and is robust across unidirectional, partially diffuse, and fully diffuse shear wave conditions.

**Results:**

We validated the accuracy of our techniques through direct comparison with phase-locked, synchronous-mode imaging in benchtop experiments using tissue-mimicking phantoms. Furthermore, we demonstrated their robustness to variations in wave orientation, excitation amplitude, and diffusivity, as confirmed by repeated measurements on the same sample under diverse conditions.

**Conclusions:**

Together, these methods may offer a more practical approach for shear wave imaging without requiring modifications to existing clinical phase-stable OCT systems.

## 1 Introduction

The biomechanical properties of tissues play a fundamental role in maintaining physiological function and are frequently implicated in disease pathogenesis. For example, the cornea accounts for approximately two-thirds of the eye’s refractive power due to its precise geometry and refractive index. Consequently, aspherical deformations caused by corneal matrix softening can lead to loss of visual acuity.^1^^,^^2^ Alterations of tissue elasticity are also increasingly recognized as a driver and indicator of cancer progression, with changes in stiffness and compliance influencing tumor growth, invasion, and metastasis.^3^ In the cardiovascular system, vulnerable atherosclerotic plaques, characterized by soft, lipid-rich cores and thin fibrous caps, are more prone to rupture, triggering thrombosis and myocardial infarction.^4^^–^^6^ These examples illustrate a need for robust, quantitative tools to assess tissue biomechanics.

One promising technique for quantifying tissue mechanics is optical coherence elastography (OCE).^7^^–^^9^ OCE is a functional extension of optical coherence tomography (OCT), an optical imaging modality that generates high-resolution, tomographic images of tissue microstructure by detecting back-scattered light.^10^^,^^11^ Utilizing interferometry, OCT produces depth-resolved, cross-sectional images with lateral resolution on the order of a few microns and axial imaging ranges up to 1 to 2 mm in tissue. OCT has already achieved widespread clinical adoption, particularly in ophthalmology, where over 30 million imaging procedures are performed annually across more than 50,000 systems worldwide.^12^ Despite its widespread utility, however, all of these existing conventional clinical OCT systems are limited in their ability to assess mechanical properties. OCE addresses this gap by employing phase-sensitive measurements with Doppler OCT^13^ to track internal tissue displacements in response to external forces, enabling the estimation of biomechanical properties through appropriate constitutive modeling.

External forces typically used in OCE span a range of approaches, including quasi-static compression,^9^^,^^14^ passive motion induced by physiological activity such as the cardiac cycle,^15^^,^^16^ and actively generated shear waves.^17^ Compared with quasi-static compression-based methods, wave-based approaches avoid the need for sensitive reference measurements by directly analyzing the propagation of shear waves through tissue.

Within shear-wave-based OCE, there are many unique manifestations that can be broadly described by two key characteristics. The first characteristic is the temporal profile of the shear wave field, which can be either transient or harmonic. The second key characteristic is the spatial makeup of the shear wave field, which can include either one, a few, or many overlapping shear waves. Unidirectional wavefront methods allow for direct measurements of group velocity, which can reveal shear wave speeds in bulk tissue.^18^^–^^21^ For more complex tissues, the Fourier transform of the wave propagation profile over time can reveal frequency-dependent relationships. In contrast, diffuse shear wave fields are generated through multiple points of excitation or through internal reflections, resulting in a superposition of many shear waves propagating in diverse directions and orientations.^22^ This multidirectional wave environment is, in theory, more compatible with structurally complex, heterogeneous, or anisotropic tissues, as it leverages boundary reflections and multiple intersecting wavefronts. The analysis of such fields is typically also conducted in three dimensions, offering a more comprehensive characterization than the one-dimensional approaches used in unidirectional wavefront methods.

Despite these advantages, generating a fully diffuse shear wave field in practice remains challenging. Locally, one or a few wavefronts tend to dominate. As a result, extensive spatial averaging, across two dimensions and on the scale of the shear wavelength, is usually required.^23^ The derivation of spherical Bessel functions used in some analyses also assumes a uniform distribution of shear waves propagating in all directions and orientations, a condition that is difficult to achieve, especially in sensitive organs such as the eye, where only a single or a few points of contact are optimal. Furthermore, as the Bessel functions imply that there is an ensemble of shear waves propagating in all directions, one would need to calculate the autocorrelation over an ensemble larger than the shear wavelength. However, this is usually not done, which goes against the assumptions used to derive the Bessel functions in the first place. Instead, we posit that a more realistic description of these conditions is a partially diffuse shear wave field, characterized by a mixture of diffuse regions and areas where one or a few unidirectional wavefronts dominate. To address this, Ormachea and Parker^24^ demonstrated that phase gradient methods may be more robust. However, their approach required fitting a plane at each spatial location, which was likely computationally intensive. It has also not yet been demonstrated with OCE. This could be because the pipeline involves multiple windowed processing steps, including phase fitting and smoothing. Although the window size used for fitting initially only requires a 1.34×1.34  mm window size, another 7×7  pixel-weighted average filtering step afterward results in a final effective window size of ∼9  mm. Given that OCE fields of view are typically comparable in size, this may explain why their method has so far been applied only in ultrasound, where imaging areas are substantially larger.

Regardless of the temporal or spatial makeup of the shear wave field, OCE typically relies on phase-sensitive detection of tissue displacement to visualize wave propagation.^13^ However, the relatively high propagation velocity of shear waves presents another challenge for OCT, which requires mechanical raster scanning of the laser beam. To overcome this, current OCE methods necessitate phase-locked acquisition, where the excitation of the shear wave must be tightly synchronized with OCT imaging. In this approach, commonly referred to as MB-mode scanning, the OCT beam remains fixed at a spatial location while the wave field is repeatedly generated. Only after capturing one or more repetitions of the wave field can the beam move to the next lateral position, where the excitation and measurement process is repeated. The full temporal evolution of the three-dimensional wave field is reconstructed by sequentially stitching together these time-resolved measurements.

MB-mode scanning has demonstrated promising results in tissue *ex vivo*. However, this scanning paradigm imposes several limitations. Most notably, any sample motion during volumetric acquisition compromises wave field reconstruction, making direct visualization of the shear wave field infeasible in dynamic or *in vivo* environments. Furthermore, because MB-mode acquisition is fundamentally based on repeated A-scans at fixed points, standard motion correction techniques developed for structural OCT and OCT angiography (OCTA), which rely on B-scan-based registration and, in some cases, lateral Nyquist sampling, are not applicable. Despite these limitations, MB-mode scanning has indeed been demonstrated in human studies *in vivo*, indicating shear wave OCE in general as a promising tool for biomechanical characterization.^21^^,^^25^^–^^27^ Still, the aforementioned limitations may explain why most studies are constrained to one-dimensional (1D) or very sparse lateral scanning, single-wavefront excitation, and limited spatial resolution and sampling. Given these factors, several groups have explored high-speed acquisition strategies, including full-field OCT, line-field OCT, and the use of ultrafast lasers (>400  kHz wavelength-sweep rate).^28^^–^^30^ Although these approaches offer improved robustness to motion and enable more reasonable imaging speeds, the requirement for specialized hardware introduces additional barriers to clinical adoption and broad translation. These ultra-fast A-line rates also come with a penalty to imaging sensitivity and Doppler signal-to-noise ratio (SNR), which are commonly addressed with volume averaging, thus negating the advantage in overall imaging speed. Furthermore, MB-mode methods are fundamentally limited by shear wave excitation frequency rather than laser speed, as they must wait for one or more whole periods of the shear wave to pass before moving to the next sampling locations. To address this limitation, continuously raster-scanned, or BM-mode imaging, has been demonstrated with a 1.6-MHz A-line rate. However, in this mode, the shear wave imaging bandwidth is limited by the B-scan rate of the system. Resonant scanners are also required to meet the multi-kilohertz B-scan rate, which results in a fixed design, making it challenging to adjust sampling density to the desired scan range and complicating acquisition of high-quality structural images alongside OCE data.^31^

Previously, we demonstrated the first fully asynchronous recovery of a semi-reverberant shear wave field using a raster-scanning configuration with a conventional A-line rate OCT system, achieving imaging of human skin *in vivo*.^32^ This approach circumvents phase-locked acquisition and offers improved motion robustness. However, it characterizes shear wave propagation in only two dimensions, necessitating line-field excitation to generate in-plane shear waves. This requirement imposes constraints on anatomical accessibility and alignment. To expand the clinical applicability of OCE, there remains a need for three-dimensional measurement and characterization of shear wave fields. Ideally, such methods would eliminate the need for the precise alignment of excitation and imaging axes as required for unidirectional wavefront methods. In addition, beyond acquisition, there remains a need for analysis techniques that remain valid across a range of shear wave field conditions while performing reliably within smaller spatial averaging windows.

In this work, we eliminated the need for in-plane shear wave excitation and achieved the first fully three-dimensional recovery of a spatially and temporally coherent shear wave field during raster scanning. We introduce this technique as asynchronous optical coherence elastography, or AsyncOCE. By leveraging asynchronous acquisition, AsyncOCE reduces the window of motion sensitivity from several seconds (a complete volume) to tens of milliseconds (a pair of B-scans), without requiring complex or specialized hardware. We also expanded the reverberant shear wave phase gradient method described in Ormachea and Parker^24^ to develop a robust estimation of shear wave number under any diffusivity regime with a computationally efficient method, reducing the window size to less than half a millimeter. Although Ormachea and Parker discussed directional filtering and believed it was not required, we found that directional filtering improved our results and enabled smaller window sizes, in particular, in areas where waves propagated in opposite directions. We termed this technique directional phase gradient analysis, or DPGA. To rigorously validate these methods, we performed benchtop experiments in tissue-mimicking phantoms, directly comparing the results to conventional phase-locked methods. Across a wide range of conditions, including varying wave orientation, scanning orientation, excitation amplitude, and levels of wave diffusivity, AsyncOCE and DPGA provided accurate and consistent shear wave speed measurements. Importantly, AsyncOCE matched the accuracy of traditional approaches while also demonstrating superior imaging speed, even with a 15-fold increase in lateral sampling density.

## 2 Materials and Methods

### 2.1 AsyncOCE for Three-Dimensional (3D) Coherent Shear Wave Recovery from Raster-Scanned Data

#### 2.1.1 Shear wave field modeling

Hereafter, vectors are denoted in bold (e.g., v) and unitary vectors as v^. We define a right-handed coordinate system where the optical axis or depth direction is z (increasing with depth), the in-plane lateral dimension is x, and the out-of-plane lateral dimension is y. The harmonic shear wave field is modeled as a superposition of one or more plane waves propagating in random directions, arising from either multiple excitation points or multiple reflections at tissue boundaries. The particle displacement S(ε,t) at a given spatial location $\varepsilon =\{\varepsilon_{x},\varepsilon_{y},\varepsilon_{z}\}$ and time t can therefore be expressed as

S(ε,t)=∑q,ln^qlsqlej2π(kn^q·ε−f0t), (1)

where n^ql denotes the direction of wave propagation, n^q is the orientation of the shear wave normal to the propagation direction, $s_{ql}$ is the amplitude, k is the wave number, and $f_{0}$ is the excitation frequency in hertz. OCT acquires depth-resolved, complex-valued measurements of backscattered light along the z-axis. To construct a 3D volume, the OCT beam is mechanically scanned by mirrors; scanning along the x-axis yields a two-dimensional (2D) B-scan, and subsequent scanning along the y-axis generates the full 3D, volumetric dataset. In this study, we employed an OCTA scanning protocol,^33^^,^^34^ which acquires just two consecutive B-scans at each y-location. This approach enables the computation of phase differences between complex-valued OCT signals and measurement of sub-pixel tissue displacement using Doppler OCT.^13^

#### 2.1.2 In-plane, 2D wave recovery theory

First, we recovered coherence along the pairs of 2D B-scans at each y-location. Previously, we have shown that due to the relatively high speed of shear waves, time cannot be considered constant within a B-scan if OCT images are acquired with conventional A-line rates and raster scanning. As a result, most OCE approaches rely on either phase-locked shear wave imaging or custom high-speed OCT systems to maintain temporal coherence. Instead of taking either of these approaches, we directly addressed the temporal variation introduced by raster scanning. For the i’th OCT frame, we accounted for the delay introduced by lateral beam motion along x by expressing time as $t_{i}+\Delta x/v_{\text{scan}}$, where Δx represents the lateral distance from the initial A-line and $v_{\text{scan}}$ is the in-plane raster scanning speed. Substituting this representation of time into the complex exponential form of the shear wave field gives

S(ε,ti)=∑q,ln^qlsqlej2π(kn^q·ε−f0(ti+Δx/vscan)), (2)

for the i’th OCT displacement frame. Compared with Eq. (1), the only difference is an additional exponential $f_{0}\Delta x/v_{\text{scan}}$ term. Following Schmidt et al.,^32^ this term reveals that the effect of raster scanning manifests as amplitude modulation, therefore allowing us to directly demodulate scanning-induced modulation and recover the original wave field. The resulting spatially coherent displacement field is complex-valued. Its real part represents the instantaneous displacement field at a single time point, whereas its phase encodes the complete harmonic shear wave field (see Sec. 2.1.4 for implementation details and equations).

#### 2.1.3 Out-of-plane, complete 3D wave recovery theory

The complex-valued displacement field resulting from in-plane shear wave recovery reveals a critical insight enabling the second step toward 3D coherence recovery and one of the key novelties presented in this work: the complex-valued displacement field at each y-location already inherently contains all the information required to reconstruct the wave field at any point in time. To produce the shear wave field at a different time point, such as t+Δt, we can directly multiply the complex displacement field by the phase term $e^{j2\pi \Delta tf_{0}}$. See Video 1 for an example of a continuous harmonic shear wave field generated from just one displacement measurement in a sample with a soft inclusion. Further details about the sample are discussed in Sec. 2.5. Given system parameters such as the OCT sampling rate and the number of samples per B-scan, we can also directly calculate Δt, the time offset, and thus the phase difference, among shear wave fields reconstructed at different out-of-plane y-locations. By cumulatively accounting for the expected phase offset at each consecutive y-location, we effectively suppressed the phase noise introduced along the y-axis due to asynchronous raster scanning. This process enables recovery of the fully coherent 3D shear wave field. In practical terms, this is equivalent to applying a linear phase ramp along the y-dimension of the volume, where the slope of the ramp corresponds to the expected phase delay among successive scan locations. See the following Sec. 2.1.4 for details and equations regarding implantation.

#### 2.1.4 Signal processing implementation for AsyncOCE

**Fig. 1:**
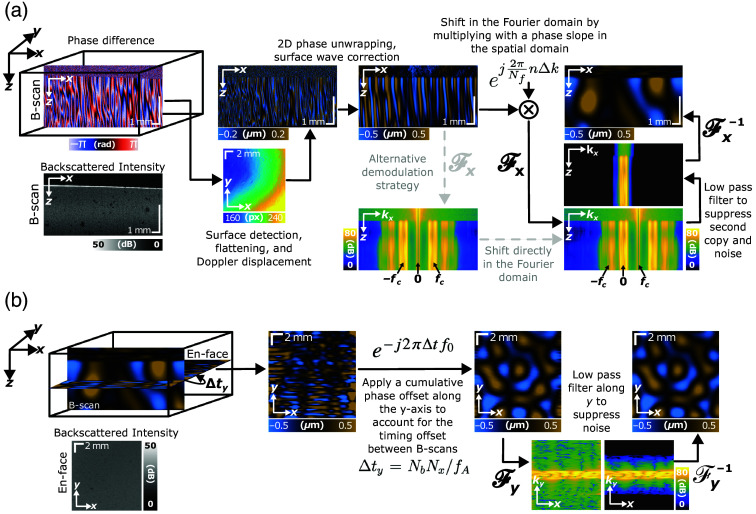
(a) First, coherence recovery is performed along individual B-scan pairs. For each B-scan, we detected the surface of the sample, flattened it, and corrected for surface waves. Then, we performed 2D phase unwrapping and demodulation in the spatial domain by multiplying with an exponential phase ramp. This conveniently allowed for non-integer values of Δk. By the discrete Fourier transform shifting property, this was equivalent to computing the Fourier transform and shifting the entire spectrum (shown in light gray). Finally, we applied a low-pass filter centered at DC to suppress the second copy of the signal and noise. The resulting displacement field at each y-location was complex valued. We computed the real part to visualize the displacement at one point in time. (b) To align the out-of-plane y-locations, we applied a cumulative phase shift along the y-dimension to each B-scan. We also applied a Blackman low-pass filter along y equal in length to the number of y-locations to suppress noise. Finally, the shear wave field was spatially and temporally coherent along all dimensions. (a) In-plane, 2D B-scan coherence recovery along the x-axis. (b) Out-of-plane, 3D volumetric coherence recovery along the y-axis.

Follow along with Fig. 1 for a detailed flow chart. After tomogram reconstruction, we computed the Doppler-measured displacements between the pair of B-scans acquired at each y-location. Then, following Ghiglia and Romero,^35^ we performed 2D phase unwrapping. We also performed surface wave correction to compensate for tissue surface motion and refractive index differences between the tissue and air.^36^

Then, raster scanning introduced in-plane amplitude modulation along the direction of scanning with a carrier frequency $f_{c}=f_{0}/v_{\text{scan}}$. Given the A-line repetition frequency $f_{A}$ and lateral scan range $R_{x}$, the carrier frequency may be rewritten as $f_{c}=f_{0}N_{f}/(f_{A}R_{x})$. Note that the raw number of A-lines acquired per B-scan (including any flyback or downtime) is defined as $N_{x}$, whereas the number of A-lines remaining after cropping or padding is defined as $N_{f}$. In our previous work,^32^ we performed demodulation by shifting the entire spectrum in the Fourier domain. In this work, however, we elected rather to apply a phase shift in the spatial domain, which more conveniently enables non-integer shifting. Essentially, by the discrete Fourier transform shift property, if $F^{-1}(X[k])=x[n]$, then

$$F^{-1}(X[k-\Delta k])=x[n]\cdot e^{j\frac{2\pi }{N_{f}}n\Delta k},$$ (3)

where Δk is the number of pixels to shift by in the Fourier domain. The interval between each pixel is then $dk=f_{s}/N_{f}=1/R_{x}$, where $f_{s}$ is the spatial sampling frequency. Given the raster-scanning modulation carrier frequency $f_{c}$, we divided by dk to compute the number of pixels to shift by. This resulted in $\Delta k=f_{0}/f_{A}\cdot N_{f}$. Therefore, the demodulation pixel amount is only dependent upon the shear wave excitation frequency and OCT A-line rate. If $S_{0}$ is the raw displacement acquired by raster scanning and $S_{d}$ is the demodulated displacement field, then

$$S_{d}[n_{x},t_{i}]=S_{0}[n_{x},t_{i}]e^{j2\pi n_{x}f_{0}/f_{A}}.$$ (4)

Finally, we computed the Fourier transform along x for each z-depth, applied a Blackman bandpass filter centered on 0 Hertz to remove noise and the mirror copy of the signal at the other carrier frequency, and applied the inverse Fourier transform. The width of the bandpass filter depended on the frequency content of the shear wave field as determined by the power spectrum. In the experiments described herein, we acquired 1536 samples along each B-scan across a 12-mm field of view and used a filter width of 25 pixels for shear waves generated at 1 kHz and 33 pixels at 2 kHz. As the shear wavelength is smaller at higher frequencies, the shear wave number is larger, and the spatial frequency content of the shear wave field will also be higher. Therefore, higher frequency shear wave fields require higher bandwidth bandpass filtering relative to lower frequencies. The specific number of pixels was chosen to maximize the signal symmetrically around the carrier frequency while rejecting high- and low-frequency noise. As the carrier frequency is determined analytically by Eq. (4), it is straightforward to determine the peak location [see Fig. 1(a) for the in-plane filtering process].

To produce the complete 3D shear wave field, 150 B-scans were acquired at 75 out-of-plane positions across 12 mm. Each out-of-plane position only requires two B-scans for displacement measurement. The resulting displacement field was complex-valued. To produce the real-valued displacement field for visualization purposes, we took the real part. For any desired phase shift ϕ, we multiplied the complex-valued field by $e^{j\phi }$ and then computed the real component after.

Now, the shear fields were spatially and temporally coherent along the B-scan xz-plane, but out-of-plane y-locations were still misaligned [see Fig. 1(b)]. To correct for this, we calculated the expected time delay among consecutive y-locations, $\Delta t_{y}=N_{b}N_{x}/f_{A}$, where $N_{b}$ is the number of repeat B-scans acquired at each y-location and $N_{x}$ is the raw number of A-lines acquired per B-scan. For a time delay of $\Delta t_{y}$ among y-locations, the phase shift required to offset it is

$$e^{-j2\pi \Delta tf_{0}}.$$ (5)

Cumulatively multiplying this term to each xz-displacement field corrected for artifactual phase jumps of the shear wave field between each y-location. As before, the resulting displacement field was complex-valued. Note that the phase shift is negative to propagate the shear wave field forward in time because we used the negative carrier frequency copy of the amplitude-modulated field for in-plane recovery. If the positive carrier frequency were used, the down-shifted field is complex-conjugated and thus a positive phase propagates forward in time, which would require the opposite phase sign for field propagation or y-location phase correction. We also applied a Blackman low-pass filter equal in length to the number of y-locations to suppress noise along the y-direction.

### 2.2 Directional Phase Gradient Analysis Estimates Local Shear Wavenumber k

Asynchronous shear wave imaging enables the recovery of spatially and temporally coherent 3D shear wave fields. However, to obtain quantitative measures of tissue elasticity, it was also necessary to extract the shear wave number k from the displacement field. Ormachea and Parker^24^ previously demonstrated estimation of k using the phase gradient of a 2D shear wave displacement field acquired with an ultra-high frame rate (>3600  frames/s) multi-element ultrasound probe. We initially adopted this approach because, in principle, it is robust across single-wave, partially diffuse, and fully diffuse shear wave environments.

However, this method assumes there is only one dominant wave within each local processing window. Similar to fully diffuse conditions, the notion of a single dominant wave within a small window is unrealistic. In practice, it is more likely that a few shear waves, each with a distinct propagation direction and phase gradient, coexist within a given region. We refer to this as partially diffuse conditions. This superposition leads to artifactual over-estimations and underestimations of k which become especially pronounced at intersections where waves propagate in opposite directions [see Fig. 4(c)]. Intuitively, we can consider the special case of two waves with identical amplitudes traveling in exactly opposite directions; these waves will create a standing wave field, which has a constant phase everywhere. Thus, the phase gradient is zero.

To address this limitation, Ormachea and Parker applied a smoothing filtering to their data, resulting in a final effective window size of ∼9  mm. Given that OCE fields of view are typically comparable in size, this may explain why their method has so far been applied only in ultrasound, where imaging areas are also larger. In their method, the shear wave number k was also estimated by fitting a two-dimensional plane to the unwrapped phase of the displacement field using linear least squares minimization. The fitting plane took the form of $k_{x}\Delta x+k_{y}\Delta y+c_{0}$, where $k_{x}$, $k_{y}$, and $c_{0}$ were the fitted parameters, and the wave number was computed from $k=\sqrt{k_{x}^{2}+k_{y}^{2}}$.

Rather than performing fitting, we modeled the harmonic shear wave field within a small window as a single plane wave, propagating along just one direction, represented by $e^{j2\pi (k_{x}x+k_{y}y)}$. Unlike previous methods, to guarantee this assumption, we performed directional filtering along x and y and computed the phase gradient for each direction individually while averaging along z. This enabled the accurate determination of the local shear wave number in OCE in much smaller fields of view. Given this formulation, the autocorrelation of the wave at a spatial offset of Δx and Δy corresponds to its product with a shifted complex conjugate, which we can simplify as

$$e^{j2\pi (k_{x}x+k_{y}y)}e^{-j2\pi (k_{x}(x+\Delta x)+k_{y}(y+\Delta y)}=e^{-j2\pi (k_{x}\Delta x+k_{y}\Delta y)}.$$ (6)

The argument, or phase, of this autocorrelation is $\phi (\Delta x,\Delta y)=2\pi (k_{x}\Delta x+k_{y}\Delta y)$. The magnitude of the gradient of ϕ(Δx,Δy) evaluated at Δx=Δy=0 results in

$$|\nabla p(\Delta x,\Delta y){|}_{\Delta x=\Delta y=0}|=\sqrt{k_{x}^{2}+k_{y}^{2}}=k,$$ (7)

with k in units of $m^{-1}$ without any optimization.

**Fig. 2:**
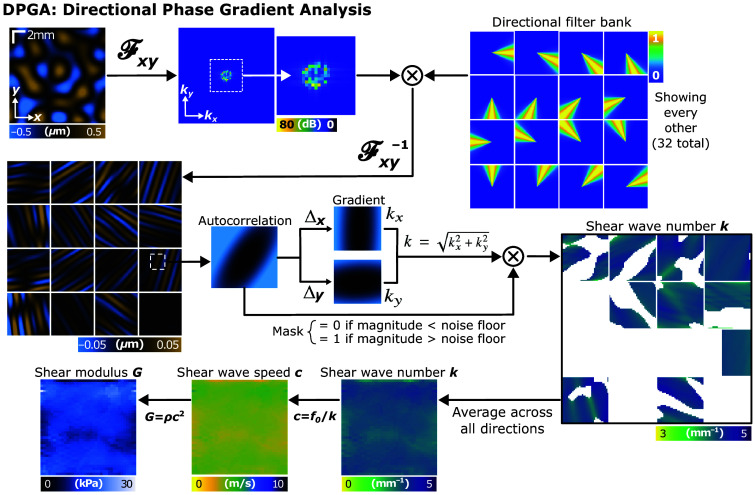
Waves traveling in opposite directions have opposite phase gradients that interfere with wave number estimation. Therefore, the first step is to perform directional filtering. We found that 32 directions were sufficient for minimizing noise. Every other filter is visualized here. The displacement field along each direction was then analyzed independently by computing the gradient of the argument of the autocorrelation within small windows. The autocorrelation was computed by taking the magnitude squared of the complex Fourier-transformed displacement field. This was particularly important for wave fields with only one or two wavefronts because the relative magnitude of waves traveling beyond the dominant directions was typically dominated by noise. Finally, shear wave numbers computed from each direction were averaged, and shear wave speed and shear modulus were computed, assuming a homogeneous isotropic sample.

Signal processing details are as follows. See Fig. 2 for a detailed flow chart. First, we applied a bank of 32 Gaussian-shaped directional filters to the displacement field and analyzed each field independently. We do not expect directional filtering to impact signal-to-noise ratio because the same filter is applied equally to the signal and noise across all spatial frequencies along a given direction. Therefore, the ratio between the signal and noise should remain the same. For each shear wave displacement field, we first computed the autocorrelation within small windows. As OCT imaging depth is relatively shallow compared with the lateral field of view, the 2D autocorrelation is computed along x and y by taking an en face window, computing the Fourier transform, squaring its magnitude, then inverse Fourier transforming. Restricting analysis to just laterally propagating Rayleigh surface waves in isotropic samples allowed us to assume that shear waves near the surface may be averaged along depth, although this analysis could also be extended to 3D in the future. To this end, we only analyzed the first few slices below the surface of each sample at a depth of 20  μm. Then, we computed the argument, or phase, of the autocorrelation and the magnitude of its gradient along x and y evaluated at Δx=Δy=0. These calculations gave directly the components of the shear wave field along each direction, allowing for direct calculation of the shear wave number $k=\sqrt{k_{x}^{2}+k_{y}^{2}}$. Across each direction, we masked the results by the displacement SNR, which was computed by taking the ratio of the measured displacement at the location of interest versus in air for each dataset because the displacement in air should be zero and therefore represents the noise floor. Then, we averaged across all directions under the assumption of laterally isotropic conditions. For the phantom data used for 3D AsyncOCE validation, the displacement tomograms had dimensions of [z,x,y]=[1000,1394,75]. Prior to computing the autocorrelation function, we downsampled the x dimension to 75 to match the y dimension. Note that AsyncOCE relies on relatively fine lateral sampling along the direction of scanning due to demodulation. This is not the case along the out-of-plane y-axis, so sampling is relaxed. Sampling for MB-mode scanning was chosen to be [x,y]=[100,75] to match the y-sampling used in AsyncOCE.

The autocorrelation function was computed with square windows of 2×2  pixels. The same window size was used for both shear wave frequencies. Given that the field of view was 12 mm, this resulted in a window size of 0.32×0.32  mm. This is smaller than the windows used in Ormachea and Parker, but our shear wave frequencies were higher, and therefore, shear wavelengths were also smaller. We also performed our processing on the complex-valued displacement field, rather than the real part typically used for wave field visualization. The complex representation preserves temporal information, enabling reconstruction of the shear wave field at any time point by applying the phase shift defined in Eq. (5). This allows the use of spatial processing windows smaller than the shear wavelength. This can also be understood by considering that the phase of the complex-valued displacement field changes by 2π radians over one shear wavelength. Therefore, the window size for DPGA processing can be significantly smaller than the wavelength. In our experiments, we used a window size approximately one-eighth of the wavelength, corresponding to a phase change of π/4=0.8 radians, well above the apparent displacement noise floor in our data (see Fig. 4). Accompanying open source code for AsyncOCE and DPGA processing is available.^37^

### 2.3 OCE System

The OCT data were acquired with a custom system, previously described in detail by Cannon et al.:^38^ briefly, the system’s laser was an optically k-clocked, phase-stable, 100-kHz VCSEL wavelength-swept laser (Thorlabs, SL131090, Newton, New Jersey, United States) operating with a center wavelength of 1310 nm. Given the role of the A-line rate for AsyncOCE signal processing, it was important to determine it with high precision, so we directly measured it and determined it to be 100.082 kHz. If the nominal value of 100 kHz were used, a spurious phase ramp along the y-axis would appear in the demodulated data. For example, given 1536 A-lines per B-scan across 150 B-scans, the difference in total theoretical scan time between 100 and 100.082 kHz for a volume adds up to 1.9 ms. This is on the same scale as an entire shear wave period. The exact A-line rate was initially calibrated by measuring a flat vibrating speaker surface at a known frequency with MB-mode phase-locked scanning. Because the surface of the speaker moves synchronously, we were able to determine residual cumulative phase offsets at each lateral location that produced a homogeneous displacement of the speaker surface. These phase offsets allowed us to calculate the true A-line rate of the system, a small but notable correction from the 100-kHz nominal specification. This technique may be useful if more advanced hardware is unavailable. As this experiment with the speaker was performed during the system characterization and debugging process, we also eventually implemented direct wavelength-sweep rate logging to monitor fluctuation over time by measuring the auxiliary output trigger signal from the frame grabber data acquisition card during background spectrum acquisition. The frame grabber signal acquisition is synchronized with the laser A-line rate via the lambda trigger from the light source; thus, the auxiliary output trigger is indicative of the true A-line rate. The frequency corresponding to the A-line rate is computed from the auxiliary output trigger signal using the Fourier transform. The rate remained stable to the nearest whole number over many weeks.

The collimated input beam had a diameter of 3.6 mm; combined with a 54-mm effective focal length objective scan lens (Thorlabs, LSM04), the system produced a diffraction-limited $e^{-2}$ diameter resolution volume of 26  μm after considering the confocal effect. The beam was raster-scanned by galvanometers (Thorlabs, GVS002) through the scan lens with a field of view up to 14.1 mm. The galvanometers were aligned to avoid introducing a phase shift during scanning. The complex fringe was acquired with polarization-diverse receivers (Advanced Fiber Resources, Zhuhai, China). The incoming signal was digitized with a digitizer (AlazarTech, ATS 9373, 4 GS/s, Pointe-Claire, Canada) with k-clocking. Shear waves were driven by a function generator through a high-performance voltage amplifier (Micromechatronics Inc., MX200, State College, Pennsylvania, United States) and a piezoelectric actuator (Digi-Key 445-181631-ND, Thief River Falls, Minnesota, United States).

### 2.4 Comparison with MB-Mode Scanning

#### 2.4.1 Experimental setup and design

**Fig. 3:**
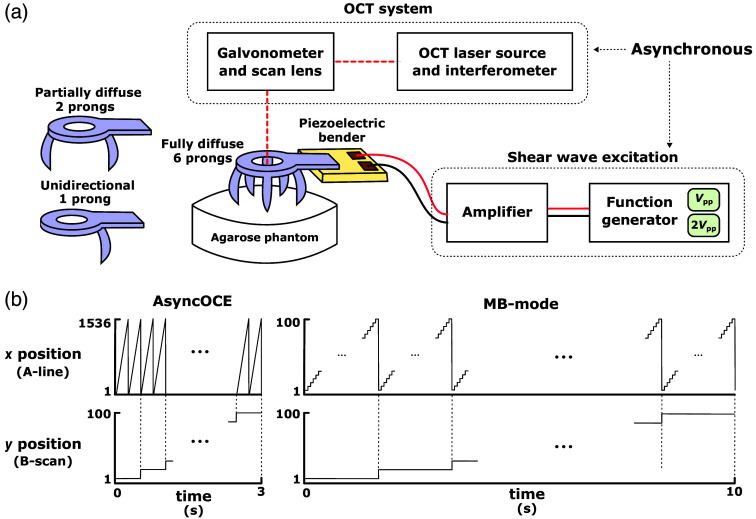
(a) OCT system and shear wave excitation mechanism are decoupled, which is the basis for the name AsyncOCE. We used 3D-printed prongs with varying points of contact to control field diffusivity. We modulated the voltage from the function generator to control the shear wave displacement amplitude and adjusted the fast and slow scanning axes of the galvanometers to control the relative orientation of the wave field to scanning. (b) Side-by-side comparison of the scanning patterns required for AsyncOCE versus MB-mode scanning. MB-mode scanning does not include a sufficiently sampled OCT structural image, which would require additional time.

To validate the AsyncOCE shear wave field recovery technique, we constructed a homogeneous aqueous tissue-mimicking phantom composed of 1.5% (w/w) agar and 0.5% (w/w) intralipid. The phantom was stored in the refrigerator prior to imaging then allowed to return to room temperature. All data were acquired within 15 min. The phantom was imaged synchronously using 1- and 2-kHz piezoelectric excitation and MB-mode scanning to obtain ground truth measurements of the shear wave field. To produce wave fields of varying amplitudes, the function generator output voltage was doubled. To modulate the degree of diffusivity in the wave field, we coupled waves into the phantoms with either 1, 2, or 6 independent 3D-printed prongs, evenly spaced at a diameter of 20 mm [Fig. 3(a)]. We also acquired each dataset twice, switching the fast and slow scanning axes in between. As in-plane and out-of-plane processing differ, the goal was to verify that our methods were ambivalent to shear wave propagation direction. In particular, in the one or two prong conditions, we wanted to verify that shear wave retrieval was robust regardless of whether shear waves were perpendicular or parallel to the B-scan plane. As the phantom itself was identical across all measurements, the measured shear wave number, k, should also remain constant at each excitation frequency. Given the temperature and hydration changes in the sample, however, shear wave speed (or modulus) may still vary slightly over time and at different excitation frequencies due to its viscosity.^39^^,^^40^

#### 2.4.2 Scanning protocols

Phase-locked volumes were acquired using MB-mode scanning [Fig. 3(b)], in which multiple A-lines were captured at a fixed lateral position before moving to the next. This was repeated at [x,y]=[100,75] lateral positions across a 12  mm×12  mm field of view. Given a nominal 100-kHz wavelength-sweep A-line rate, each shear wave period corresponded to 50 A-lines for 2-kHz excitation and 100 A-lines for 1 kHz. To capture two complete wave periods for averaging, we acquired 100 and 200 A-lines for 2- and 1-kHz excitation, respectively, resulting in total acquisition times of 7.5 and 15 s. As mentioned above, because we later determined that the experimental A-line rate was actually 100.082 kHz, we also applied a phase offset in post-processing to the MB-mode scans at each location accordingly to achieve ground-truth temporal coherence.

For AsyncOCE imaging, we acquired 1536 A-lines per B-scan and performed 2 repeat B-scans at each y-location, across 75 y-locations. The flyback consisted of the first 142 A-lines from each B-scan. These A-lines are cropped out when considering spatial sampling frequency $f_{s}$, but do account for the time delay Δt among y-locations. This resulted in a total acquisition time of 2.3 s for both 1- and 2-kHz excitation. Because MB-mode scanning time scales linearly with the period of the shear wave, it becomes increasingly time-consuming at lower frequencies. In contrast, AsyncOCE acquisition time is independent of wave period, yielding a 3- to 6-fold reduction in imaging time along with a 15-fold improvement in lateral sampling density, yielding high-quality structural images without the need for additional scanning.

### 2.5 Imaging a Heterogeneous Sample and Comparison with RevOCE

#### 2.5.1 Experimental setup

We also constructed and imaged a heterogeneous tissue-mimicking phantom. The sample was composed of 1.25% (w/w) agarose and 0.5% (w/w) intralipid. A 4.5-mm circular punch was used to remove material from the center, into which a softer phantom mixture composed of 0.75% (w/w) agarose and 0.5% (w/w) intralipid was poured and allowed to set at room temperature. To modulate the degree of diffusivity in the wave field, we coupled waves into the phantoms with either 1, 2, or 6 independent 3D-printed prongs, evenly spaced at a diameter of 20 mm [Fig. 3(a)]. We also applied both 1- and 2-kHz piezoelectric excitation under each shear wave field diffusivity condition to enable observation of the effect of shear wavelength on OCE processing. We also performed DPGA with and without directional filtering to observe the impact of directional filtering in a heterogeneous sample. Similar to the post-processing that is performed in Ormachea and Parker, we applied a Gaussian smoothing filter with σ equal to 2 pixels to the results obtained without directional filtering. This corresponded to a full-width half-maximum of ∼5  pixels.

#### 2.5.2 RevOCE implementation

Following Parker et al.,^22^ we implemented reverberant OCE (RevOCE) for comparison. First, the 2D autocorrelation of the displacement field was computed in small windows along the x- and y-axes. Then, we took the normalized real part of the 2D autocorrelation and computed the azimuthal average with the Radon transform. Finally, we fit the 1D result to the following sum of spherical Bessel functions:

$$B_{S_{z}S_{z}}(\Delta \varepsilon_{x,y},\Delta t)=j_{0}(k\Delta \varepsilon_{x,y})-\frac{j_{1}(k\Delta \varepsilon_{x,y})}{k\Delta \varepsilon_{x,y}},$$ (8)

where $S_{z}$ is the displacement along the sensor axis, $\varepsilon_{x}$ is the B-scan axis along which the beam is scanned, and Δε represents the difference along a given dimension for which the autocorrelation is calculated. RevOCE was applied to the same down-sampled 75×75  pixel en face displacement field as DGPA. We used windows of 6×6  pixels to compute the autocorrelation. This was the smallest window size that yielded reasonably consistent results. We used the second zero crossing to fit the spherical Bessel functions. If the second zero crossing was not within the the maximum displacement difference $\Delta \varepsilon_{x,y}$, we used the entire window to fit the spherical Bessel functions. As the resulting shear wave maps from DPGA were 35×35  pixels, the resulting RevOCE map was upsampled to match this size. We also applied a Gaussian smoothing filter with σ equal to two windows to the upsampled RevOCE results.

## 3 Results

### 3.1 AsyncOCE Validated by MB-Mode Scanning

**Fig. 4:**
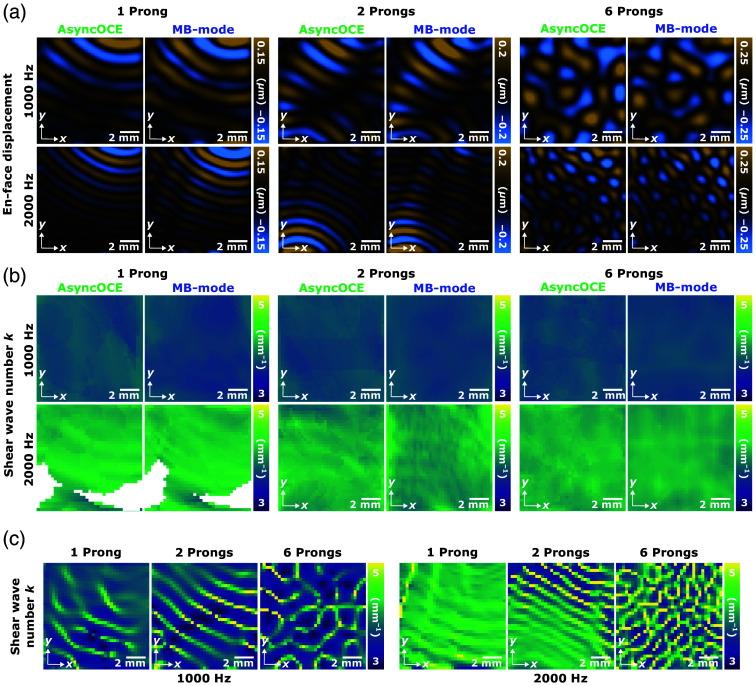
(a) En face displacement fields are shown across varying conditions. Visually, AsyncOCE retrieves the same wave fields as MB-mode imaging. (b) Therefore, it is a promising sign that the shear wave numbers would also match. The masked data for one prong at 2 kHz are due to the low displacement magnitude. Importantly, DPGA is impervious to wave conditions. Whether there is one wave, two waves, or a diffuse field of many waves, DPGA can recover reasonable shear wave number measurements from the entire spatially and temporally coherent shear wave field. A quantitative analysis of these results is shown later in Fig. 5. (c) DPGA results without directional filtering show the importance of distinguishing among waves traveling along different directions. The same exact window size (2×2  pixels) and processing steps as the full DPGA were applied to produce these results, with the only exception being skipping the directional filtering. (b) DPGA. (c) Conventional analysis (no directional filtering).

**Fig. 5:**
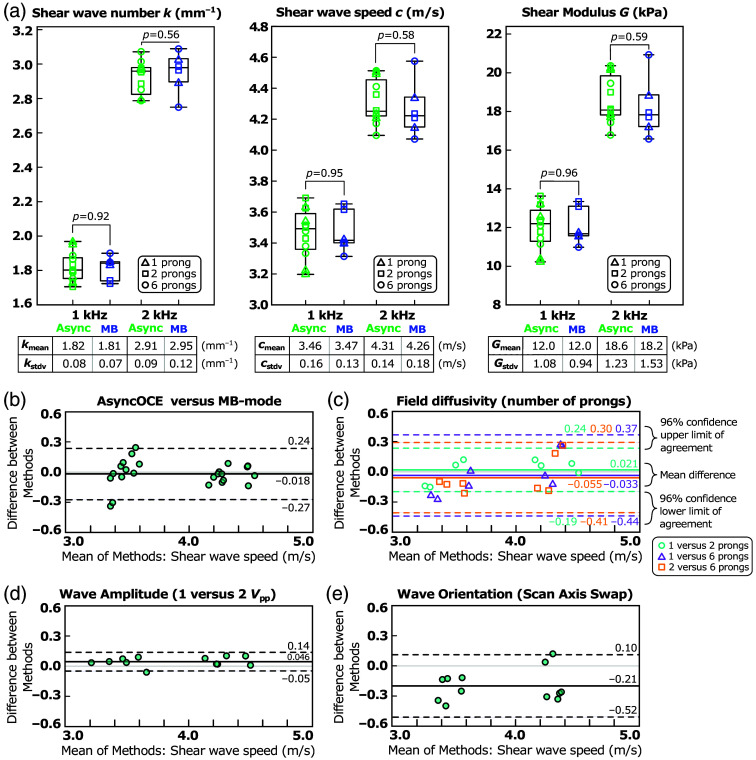
(a) Quantitative comparison of shear wave number, speed, and shear modulus across various frequencies and wave diffusivity, demonstrating strong agreement between AsyncOCE and phase-locked MB-mode scanning. Triangular symbols represent unidirectional, single-prong shear wave excitation. Square symbols represent partially diffuse, two-prong shear wave excitation. Circular symbols represent diffuse, six-prong shear wave excitation. There are four data points for each shear wave excitation condition, all acquired on the same phantom. They represent all four combinations of the two shear wave amplitudes and two scanning orientations. (b) Bland–Altman plot showing the difference in measurements among methods versus their mean. Limits of agreement include zero, indicating no significant bias and strong concordance among techniques. Data with a mean shear wave speed between 3 and 4  m/s are from 1 kHz excitation, and the data with a mean shear wave speed between 4 and 5  m/s are from 2-kHz excitation. (c) Comparable results are observed when varying the number of prongs used to generate shear waves. (d) Shear wave amplitude has minimal effect on the measured shear wave speed. (e) Shear wave speeds remain consistent regardless of whether galvanometer scanning is performed parallel or perpendicular to the wave propagation direction and across different levels of diffusivity.

We compared shear wave field measurements obtained using AsyncOCE to the same measurements acquired using phase-locked, MB-mode scanning. Both methods employed the same directional phase gradient analysis, or DPGA, to calculate shear wave number, shear wave speed, and shear modulus; however, the method of shear wave field acquisition differed. Representative results across different frequencies and levels of wave diffusivity are presented in Fig. 4, with a quantitative analysis following in Fig. 5. T-tests revealed no significant difference between the datasets from AsyncOCE and MB-mode imaging. To further evaluate agreement among methods, we used a Bland–Altman plot [Fig. 5(b)], which displays the difference among methods (vertical axis) against their mean (horizontal axis). This is a pairwise analysis, so results from the same excitation conditions were compared (the same frequency, the same number of prongs, and the same excitation amplitude). If the two methods yielded different results, the limits of agreement would not include zero. However, the analysis indicates that AsyncOCE and MB-mode imaging produce statistically indistinguishable measurements. We also showed representative results from phase gradient analysis without directional filtering. As anticipated, measurements are most distorted where there is a superposition of two or more waves traveling along different directions due to opposing phase gradients. We hypothesize that this is why conventional phase gradient methods without directional filtering used large averaging windows, much greater than the shear wavelength, to smooth out these artifacts. With directional filtering, we can achieve measurement windows which are smaller than the shear wavelength.

### 3.2 AsyncOCE and DPGA Robustness

We also performed Bland–Altman analyses to evaluate the consistency of measurements across different numbers of prongs used for shear wave excitation. As the Bland-Altman analysis is inherently pairwise, only two conditions can be compared at a time. Comparisons were made using the same excitation frequency and wave amplitude. Across all conditions, the phase gradient method yielded statistically indistinguishable measurements, regardless of wave field diffusivity, demonstrating its robustness to varying shear wave field properties.

Because shear wave speed primarily depends on the shear modulus and is theoretically independent of wave amplitude, we expected that variations in amplitude would have minimal impact on the measured speed. This expectation is confirmed by the results shown in Fig. 5(d). These findings help relax potential constraints on AsyncOCE imaging *in vivo*, as the processing is robust to variations in shear wave coupling efficiency, thereby accommodating expected operator variability.

Finally, the orientation of the scanning axis determines whether the waves are sampled parallel or perpendicular to their propagation direction. This is particularly relevant in unidirectional wave fields, where our previously published method could not distinguish between in-plane and out-of-plane wave propagation. In contrast, AsyncOCE can recover full 3D wave fields and is therefore robust to scanning orientation, as demonstrated in Fig. 5(e).

### 3.3 AsyncOCE and DPGA in a Heterogeneous Sample

**Fig. 6:**
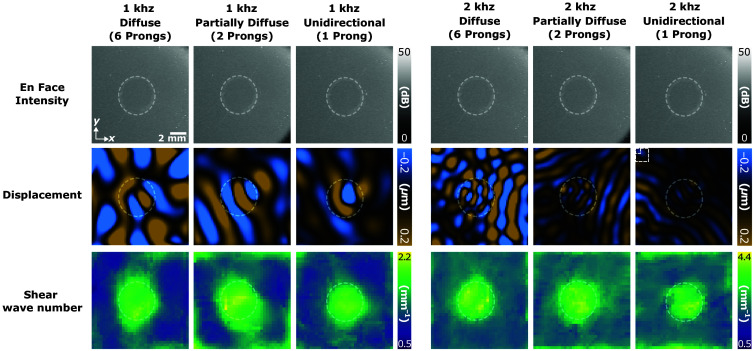
Same heterogeneous sample was imaged under varying shear wave excitation frequencies and numbers of prongs; 1, 2, or 6 prongs generated unidirectional, partially diffuse, and fully diffuse shear wave fields, respectively. The first row shows en face intensity images averaged over a shallow axial region near the surface. As identical concentrations of intralipid were used in both regions, the backscattered intensity appears similar, making visual differentiation difficult. The white dotted line indicates the location of the soft inclusion. The second row displays the recovered displacement fields. AsyncOCE consistently reveals shear waves with shorter wavelengths within the soft inclusion, regardless of field diffusivity or excitation frequency. The third row shows shear wave number maps obtained using DPGA. Visually, differences among the levels of field diffusivity are minimal. All images have a field of view of 12×12  mm (Video 1, MOV, 7.67 MB [URL: https://doi.org/10.1117/1.JBO.30.12.124506.s1]).

**Fig. 7:**
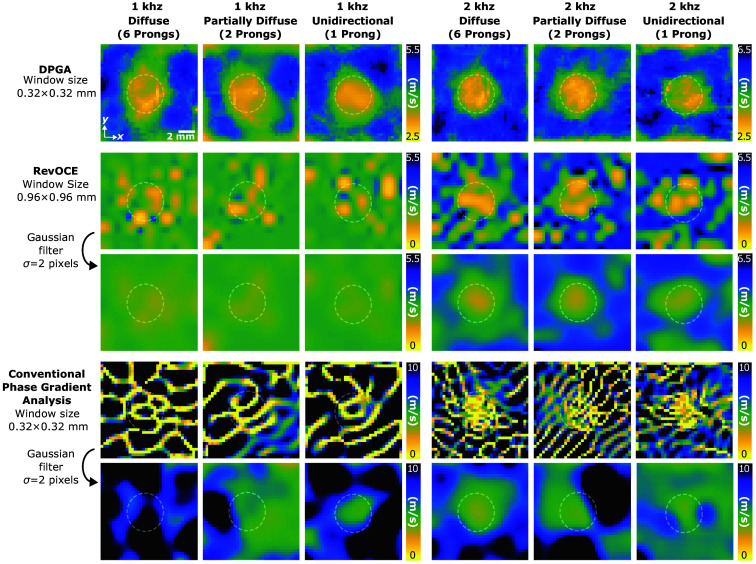
Same sample containing a soft inclusion was processed using DPGA, RevOCE, and conventional phase gradient analysis without directional filtering. Shear wave fields were induced using prongs configured to generate unidirectional, partially diffuse, and diffuse wave patterns. Shear wave speed maps are shown for each post-processing method across different levels of field diffusivity and excitation frequencies. Due to the high variability in results from the alternative methods, note the expanded color bar ranges used for those images. Gaussian-smoothed versions of the RevOCE and phase gradient results are shown below each corresponding image. All images share a 12  mm×12  mm field of view.

AsyncOCE and DPGA were also applied to a heterogeneous phantom consisting of a stiff background and a soft, circular inclusion. As shown in Fig. 6, the soft and stiff regions are difficult to distinguish in the intensity images, highlighting the need for elastography. In the asynchronously recovered shear wave displacement fields, shorter shear wavelengths were observed within the soft inclusion, as expected. See Video 1 for the animated harmonic shear wave field generated by 2-kHz excitation and six prongs. Note that this video is generated from just two repeat B-scans at each y-location, therefore resulting in just one displacement measurement. As the shear wavelength is generally inversely proportional to excitation frequency, the 2-kHz excitation produced smaller wavelengths compared with 1 kHz. Given these displacement fields, DPGA revealed a clear contrast between the soft and stiff regions, regardless of the shear wave field’s level of diffusivity. Differences in shear wave speed, as shown in Fig. 7, between the two excitation frequencies are likely influenced by viscosity effects, consistent with observations in the homogeneous samples. Bright edges along the outside border of each image are due to edge artifacts introduced during image processing.

Figure 7 compares DPGA with both RevOCE processing and phase gradient analysis without directional filtering. Due to noise in the raw outputs, Gaussian filtering was applied to both alternative methods. Although these methods detected some contrast, their performance was limited, particularly when the shear wavelength was comparable to or larger than the feature size. As shown in Fig. 6, the displacement fields indicate that the diameter of the soft inclusion is on the same scale as the shear wavelength at 1 kHz while containing a few more wavelengths at 2 kHz. As a result, both methods performed better at higher excitation frequencies. The same effect applies to the size of the processing window, which was kept constant across both excitation frequencies.

RevOCE tended to underestimate shear wave speed when the shear wavelength exceeded the window size, as seen in the 1-kHz results. At 2 kHz, RevOCE more clearly distinguished the inclusion. Phase gradient analysis without directional filtering, on the other hand, was more susceptible to artifacts from wave interference. At 1 kHz, its best performance occurred under unidirectional excitation, likely due to fewer interfering waves. Interestingly, at 2 kHz, when the shear wavelength was small relative to the smoothing window, better results were achieved using the diffuse field. Because temperature is known to affect the viscoelastic properties of agarose,^39^ we did not attempt to quantify or directly compare resolution across methods. In particular, these phantoms were created by pouring a warm agarose mixture into a cavity in the pre-cooled stiff background, potentially introducing boundary artifacts due to uneven heating and cooling. A rigorous, quantitative comparison of resolution under varying shear wave conditions and feature sizes remains an area for future investigation.

## 4 Discussion

We introduced AsyncOCE, a framework for shear wave-based OCE that enables asynchronous acquisition and reconstruction of fully three-dimensional temporally and spatially coherent shear wave fields using standard OCT hardware and scanning protocols. Unlike conventional phase-locked MB-mode OCE techniques, AsyncOCE offers faster imaging speeds and reduced sensitivity to motion, without requiring specialized or ultrafast hardware. This flexibility simplifies the imaging workflow and broadens the potential for clinical and dynamic applications, where motion and acquisition time constraints typically limit OCE’s promising capabilities.

Our validation experiments demonstrated that AsyncOCE provides shear wave speed measurements consistent with those obtained from gold-standard phase-locked MB-mode OCE, across a wide range of excitation amplitudes, wave orientations, scanning orientations, and levels of shear wave diffusivity. These results confirm that AsyncOCE maintains quantitative accuracy even under variable and non-ideal conditions, highlighting its robustness and reliability for practical use. These properties have important implications for imaging *in vivo*, as the method is robust to variability in how shear wave excitation is coupled into the tissue. This reduces the need for precise control over coupling conditions and shortens imaging time, greatly simplifying the implementation of AsyncOCE *in vivo*.

In addition to the acquisition framework, we introduced DPGA, an autocorrelation-based phase gradient method to estimate shear wave number from complex-valued displacement fields. Importantly, this technique preserves directional information while maintaining robustness in the presence of both unidirectional and partially diffuse wave fields, a common scenario in biological tissues where fully diffuse conditions are difficult to achieve in practice. It is also important to note that directional filtering has already been established in other realms of harmonic shear wave elastography, such as in ultrasound^41^ and magnetic resonance^42^^,^^43^ imaging, pointing to it as a reasonable and potentially critical processing step, especially depending on the shear wave excitation conditions and processing framework.

Finally, we acquired measurements in a heterogeneous phantom containing a soft inclusion. Under various conditions, both AsyncOCE and DPGA demonstrated a clear contrast between the soft and stiff regions. We also compared these results to alternative processing methods applied to the same shear wave field captured by AsyncOCE, including RevOCE and phase gradient analysis without directional filtering. Although these methods also revealed contrast, their performance varied between high- and low-frequency shear wave excitations. This highlights the advantage of DPGA in scenarios where the shear wavelength is comparable to the feature size of interest. Further quantitative studies are needed to validate these comparisons.

Given this new framework, there are several opportunities for future directions. First, the current implementation relies on a harmonic excitation with just one frequency at a time. Given the amplitude modulation induced by scanning, multiplexing multiple frequencies could enable nonlinear tissue characterization.^44^ To limit the scope of this work, our analysis also focused on depth-averaged Rayleigh surface waves in homogeneous phantoms. Applications in layered, guided, heterogeneous, or anisotropic tissues will require further development of the wave model and inversion algorithms. Regardless, AsyncOCE’s ability to robustly recover the shear wave field in 3D lays a strong foundation for such future advancements.

## 5 Conclusion

We introduced asynchronous optical coherence elastography (AsyncOCE), a method for recovering the full three-dimensional, temporally coherent shear wave field using conventional raster-scanning OCT systems. By modeling the effects of asynchronous acquisition as amplitude modulation, recovering out-of-plane coherence with phase delays, and eliminating the need for phase-locked imaging of harmonic shear waves, AsyncOCE addresses longstanding barriers to performing elastography *in vivo*, particularly in dynamic environments where motion renders traditional approach more challenging. Through phantom experiments, we demonstrated that AsyncOCE yields accurate shear wave speed measurements across a range of shear wave conditions, including variations in amplitude, directionality, and diffusivity. By adapting the phase gradient method to use the autocorrelation, we also enabled quantitative recovery of shear wave number without relying on plane-fitting. Validation against conventional phase-locked methods confirmed that AsyncOCE maintains quantitative accuracy while offering key advantages in speed and clinical feasibility. Altogether, this work lays the foundation for translating OCE into new clinical domains.

## Supplementary Material

10.1117/1.JBO.30.12.124506.s1

## Data Availability

The data may be obtained from the authors upon reasonable request. Sample processing scripts and test data are available at https://github.com/gingerschmidt/AsyncOCE.^37^
